# Malaria risk factors in northern Namibia: The importance of occupation, age and mobility in characterizing high-risk populations

**DOI:** 10.1371/journal.pone.0252690

**Published:** 2021-06-25

**Authors:** Jennifer L. Smith, Davis Mumbengegwi, Erastus Haindongo, Carmen Cueto, Kathryn W. Roberts, Roly Gosling, Petrina Uusiku, Immo Kleinschmidt, Adam Bennett, Hugh J. Sturrock

**Affiliations:** 1 Malaria Elimination Initiative, Global Health Group, University of California San Francisco (UCSF), San Francisco, California, United States of America; 2 Multidisciplinary Research Centre, University of Namibia, Windhoek, Namibia; 3 School of Medicine, Faculty of Health Sciences, University of Namibia, Windhoek, Namibia; 4 National Ministry of Health and Social Services, Windhoek, Namibia; 5 Department of Infectious Disease Epidemiology, London School of Hygiene and Tropical Medicine, London, United Kingdom; 6 Department of Immunology and Infection, London School of Hygiene and Tropical Medicine, London, United Kingdom; Freelance Consultant, Myanmar, MYANMAR

## Abstract

In areas of low and unstable transmission, malaria cases occur in populations with lower access to malaria services and interventions, and in groups with specific malaria risk exposures often away from the household. In support of the Namibian National Vector Borne Disease Program’s drive to better target interventions based upon risk, we implemented a health facility-based case control study aimed to identify risk factors for symptomatic malaria in Zambezi Region, northern Namibia. A total of 770 febrile individuals reporting to 6 health facilities and testing positive by rapid diagnostic test (RDT) between February 2015 and April 2016 were recruited as cases; 641 febrile individuals testing negative by RDT at the same health facilities through June 2016 were recruited as controls. Data on socio-demographics, housing construction, overnight travel, use of malaria prevention and outdoor behaviors at night were collected through interview and recorded on a tablet-based questionnaire. Remotely-sensed environmental data were extracted for geo-located village residence locations. Multivariable logistic regression was conducted to identify risk factors and latent class analyses (LCA) used to identify and characterize high-risk subgroups. The majority of participants (87% of cases and 69% of controls) were recruited during the 2016 transmission season, an outbreak year in Southern Africa. After adjustment, cases were more likely to be cattle herders (Adjusted Odds Ratio (aOR): 4.46 95%CI 1.05–18.96), members of the police or other security personnel (aOR: 4.60 95%CI: 1.16–18.16), and pensioners/unemployed persons (aOR: 2.25 95%CI 1.24–4.08), compared to agricultural workers (most common category). Children (aOR 2.28 95%CI 1.13–4.59) and self-identified students were at higher risk of malaria (aOR: 4.32 95%CI 2.31–8.10). Other actionable risk factors for malaria included housing and behavioral characteristics, including traditional home construction and sleeping in an open structure (versus modern structure: aOR: 2.01 95%CI 1.45–2.79 and aOR: 4.76 95%CI: 2.14–10.57); cross border travel in the prior 30 days (aOR: 10.55 95%CI 2.94–37.84); and outdoor agricultural work at night (aOR: 2.09 95%CI 1.12–3.87). Malaria preventive activities were all protective and included personal use of an insecticide treated net (ITN) (aOR: 0.61 95%CI 0.42–0.87), adequate household ITN coverage (aOR: 0.63 95%CI 0.42–0.94), and household indoor residual spraying (IRS) in the past year (versus never sprayed: (aOR: 0.63 95%CI 0.44–0.90). A number of environmental factors were associated with increased risk of malaria, including lower temperatures, higher rainfall and increased vegetation for the 30 days prior to diagnosis and residing more than 5 minutes from a health facility. LCA identified six classes of cases, with class membership strongly correlated with occupation, age and select behavioral risk factors. Use of ITNs and IRS coverage was similarly low across classes. For malaria elimination these high-risk groups will need targeted and tailored intervention strategies, for example, by implementing alternative delivery methods of interventions through schools and worksites, as well as the use of specific interventions that address outdoor transmission.

## Background

Namibia is a low malaria transmission country in southern Africa that has successfully reduced the burden of malaria over the past decade through the large-scale deployment of indoor residual spraying (IRS), distribution and use of long-lasting insecticide-treated bed nets (ITNs), increased use of rapid diagnostic tests (RDTs) and treatment with artemisinin-based combination therapy (ACT) [1]. The country aimed to eliminate malaria by 2020, but has experienced a number of outbreaks of malaria in northern Namibia since 2016 [2]. New approaches are urgently needed to understand and address persistent low levels of transmission and prevent seasonal epidemics of malaria.

In low malaria transmission settings, it is common to observe a shift in epidemiology towards older age groups as well as clustering of infections by location, time and within sub-populations with shared risk factors for infection [3]. Previous studies in North West and central Namibia established the importance of malaria importation and local risk factors in determining patterns of disease, including spatial clustering of infection and cross-border travel [4, 5]. An understanding of spatial clustering has helped to inform the design of geographically targeted interventions such as reactive case detection (RACD) [5, 6], reactive focal mass drug administration (rfMDA) [7] and foci investigations. However, the effectiveness of these strategies will be limited in populations where treatment-seeking is low, asymptomatic infection is more common, the population is highly mobile or residents are frequently absent or large numbers of infections are among non-resident populations. An evidence-based and transparent approach to describing local populations at high risk of infection can provide critical information on coverage gaps, mobility patterns and drivers of infection to inform proactive and tailored intervention strategies.

Malaria Indicator Surveys (MIS) and other cross-sectional studies are often used to provide malaria control programs with useful information on malaria associated risk factors. In low transmission settings such as Namibia, an MIS can identify asymptomatic infections but without more sensitive methods to measure exposure (such as serology) is unlikely to provide useful information to the national malaria control program (NMCP) on new malaria risk factors, because of the low chance of detecting malaria in the community and high expense [4, 8–11]. Case-control methodologies are a classic epidemiological approach used in rapid outbreak investigations, including for malaria [12–18] and for investigation of rare diseases. As such, this approach is particularly well suited to risk-factor identification in malaria elimination settings, where cases are few and cross-sectional surveys are likely to be underpowered. Detailed information on behavioral and occupational exposures, along with standard questions on use of malaria prevention tools, can characterize important individual risk factors and intervention targets. A remaining challenge is to understand how risk factors co-occur and translate results into actionable risk profiles for specific populations. Latent class analysis (LCA) is a type of mixture modeling that assumes the population consists of unknown sub-populations (latent classes) that differ in their mix of included variables and provides the ability to identify these latent classes. LCA has been used to characterize patterns of multi-risk profiles in relation to HIV and HCV [19–21] and can offer insights to optimize implementation of preventive measures.

This paper presents results from a case-control study in Zambezi Region, Namibia, which investigated local risk factors for malaria and overlap between behavioral and epidemiological risk factors, in order to profile distinct high risk populations and hence identify potential targets for interventions.

## Methods

### Study setting

The study was undertaken in Zambezi Region, located in the north-east corner of Namibia, and bordered by Angola, Zambia, and Botswana. Historically, it has some of the highest rates of malaria incidence in Namibia. From 2004 to 2015, the annual parasite incidence in Zambezi fell from over 600 to 17.1 cases per 1000 [22]. Zambezi Region is geographically diverse, encompassing floodplains, woodlands and wetlands along the Zambezi River. The area is largely agricultural, rural and poor, with the 2011 census estimating that nearly a third (30.2%) of the population is engaged in subsistence agriculture [23]. Malaria transmission is highly seasonal and typically occurs between November and June, with peak case numbers occurring between February and April corresponding to the rainy season. Reported malaria cases are almost all due to *Plasmodium falciparum* and the most common malaria vector species is *Anopheles arabiensis* [24, 25].

**Fig 1:**
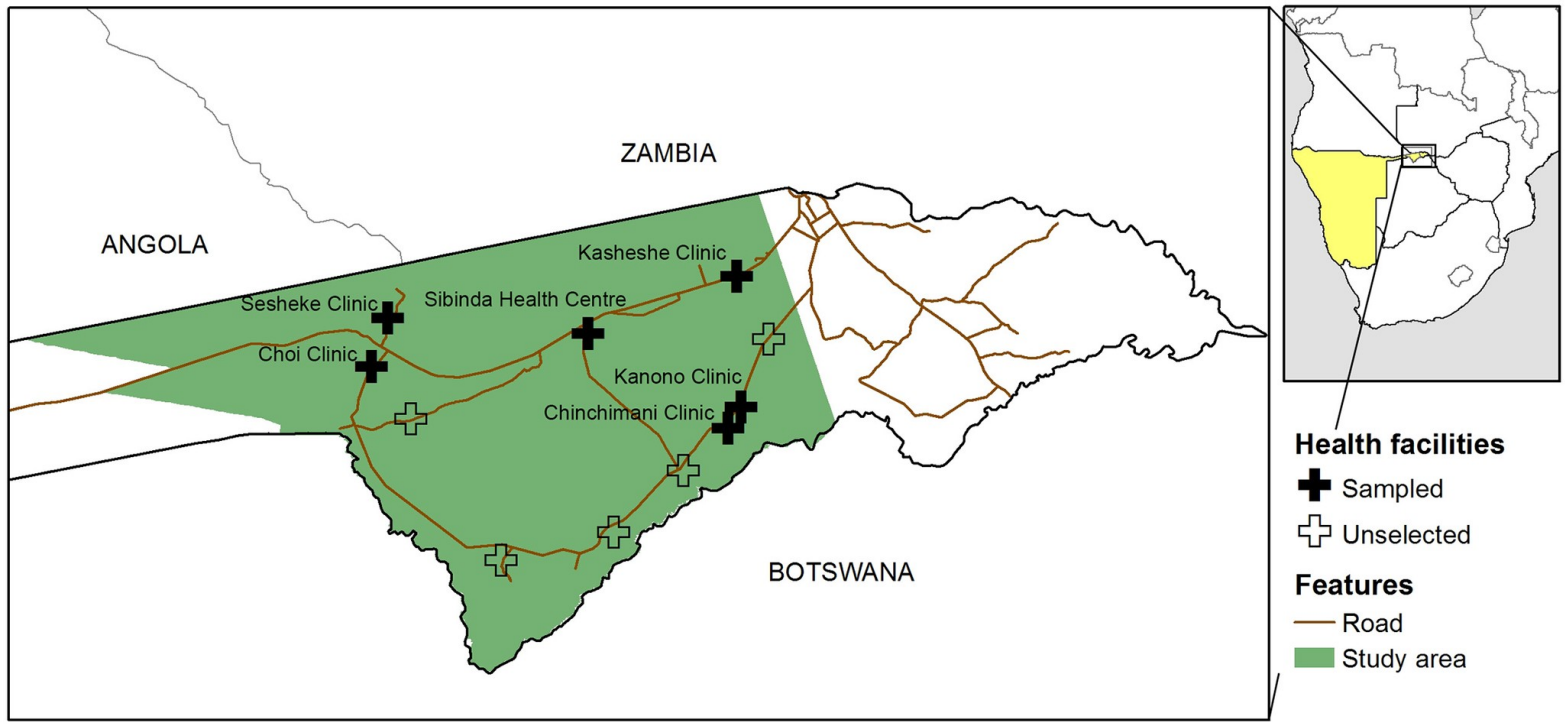
Location of six selected health facilities within the larger study area, Zambezi Region, Namibia.

The study area encompassed a geographical area of about 9500 km^2^ in Western Zambezi region and was the site for a number of baseline epidemiological studies prior to the start of a large trial in January 2016 to evaluate the impact of targeted intervention strategies on malaria transmission [26] (Fig 1). The study area borders with Angola, Botswana, and Zambia and includes one official border crossing with Zambia. A total of 12 health facilities provide primary care services, including free malaria testing and treatment for Namibians, to the approximately 32,500 residents.

### Study design

This was a health facility based, case-control study conducted over two malaria transmission seasons. Case and control recruitment was carried out between February 2015 and April 2016, with control recruitment extended through June 2016 to reach the desired sample size. Eligibility criteria for all participants included current or prior history of fever within 48 hours and valid test for malaria by the Carestart^™^ Malaria HRP-2/pLDH (Pf/pan) Combo rapid diagnostic test (RDT) at one of six randomly selected health facilities. The malaria case definition included all passively detected cases of malaria confirmed by RDT. Controls were selected from individuals presenting at the same health facilities as those who tested negative for malaria by RDT. Individuals who reported taking malaria prophylaxis or treatment within the past 14 days or having a previous diagnosis of malaria within the past 30 days were ineligible.

Controls were frequency matched to incident cases by gender and within broad age categories (less than five years, five to 14 years, 15 to 59 years and 60 years and above). Both age and gender are known to be important risk factors for malaria and frequency matching ensures sufficient power to investigate specific behavioral risk factors within populations that are generally underrepresented at health facilities (i.e. adult males). Numbers of controls by age and gender were allocated to health facilities for recruitment based on probability proportional to the size (PPS) of the population in the catchment. This ensured that the distribution of controls matched the underlying population distribution for optimal identification of malaria hotspots and risk mapping. Catchment populations were calculated by aggregating a gridded population surface for 2015 from WorldPop [27] within geographical catchment areas, as defined by Alegana et al [28]. At each health facility, the first incident control that matched an existing recruitment profile was recruited into the study.

### Data collection

Following routine diagnosis by health facility staff based on malaria RDT result, research assistants based at health facilities screened individuals for eligibility as a case or a control. Consenting participants were interviewed using a tablet-based questionnaire in Open Data Kit (ODK) and asked a set of standardized, pre-tested questions on demographics, socio-economic variables, household construction, use of malaria prevention measures, overnight travel outside their main residence in the past 30 days and outdoor activities during sundown and sunup in the prior two weeks.

### Data processing and definitions

Questionnaire data were uploaded from the tablet into a secured centralized server on a daily basis and used to generate control profiles for recruitment, based on incident case demographics. Control profiles were allocated to facilities for recruitment using probability proportional to size (PPS) of the catchment population. Regular data checks and processing were carried out in R version 3.4 in order to track recruitment, as well as identify and correct any data entry errors in a timely manner.

A categorical variable was used to indicate calendar quarters and control for differing distributions of case and controls recruited into the study. For descriptive summaries, a binary variable was created to distinguish individuals recruited early in the transmission season, when cumulative cases were below 20% of annual total (November to February). Occupational variables were grouped according to broad categories and combined if numbers represented less than 5% of cases or controls. Where individuals declined to answer a question or a low proportion (<5%) responded “Don’t know”, responses were recategorized as missing and dropped from logistic regression analyses. Selected outdoor occupations were identified *a priori* as potentially high risk (cattle herders, agricultural workers, police and security guards) based on night-time exposures discussed during formative research and were kept as separate categories in exploratory analyses. A principal components analysis (PCA) was conducted to capture variation among self-reported household assets, including electronic goods, electricity, personal effects and transportation related assets. The first component was included in the model as a socioeconomic measure, in addition to water source, latrine type and education. Following Tusting et al [29], a housing variable was generated to distinguish traditional homes (ones with mud walls, thatched roofs and earth floors) from modern homes [29]. Tents and structures with open walls were considered as separate categories. In addition, binary variables were generated for sleeping outdoors in the past two weeks and sleeping in a room with open eaves.

Travel destinations and village residences were geo-referenced to a specific longitude and latitude using a range of sources. A comprehensive database of village locations in Zambezi Region generated during a complete enumeration of all households within the study area in early 2015 was used as the gold standard [30]. Locations outside of the study area were geo-referenced using the 2011 census database and electronic gazetteers, including GeoNet Names Server [31], Google Earth [32], and Falling grain [33]. The majority of cases (94.2%) and controls (96.4%) were successfully geolocated to a village residence with coordinates and 87.6% of trip destinations. The remaining locations were assigned to a constituency. Domestic travel destinations were categorized as having “high” risk of malaria transmission if they were located in areas of Zambezi Region where the relative case burden within the encompassing health facility catchment area was known to be above 10% of the regional total in 2016. In the absence of sub-regional data, areas located in endemic northern regions were also classified as “high” compared to other areas of Namibia. An individual’s most recent trip was used to characterize travel duration. Migration within the past six months from domestic or international locations was captured as a categorical variable.

Satellite-derived environmental data were extracted from Google Earth Engine [34] for each participant’s village location for spatial analysis and inclusion in logistic regression models, including elevation, land cover type, distance to permanent water bodies and average measures of land surface temperature (LST), total rainfall and enhanced vegetation index (EVI; a proxy for vegetation coverage) for the 30 days prior to diagnosis. A summary of the source of each of these data sets and the resolution available is included in S1 Table.

### Statistical analysis

Assuming a probability of exposure among controls of 0.1 and an odds of acquiring malaria in exposed subjects relative to unexposed subjects of 2.25, a minimum target of 221 cases and 442 controls was required to reject the null hypothesis that there is no difference between cases and controls (i.e. odds ratio of 1), with 80% power and an α of 0.05.

STATA 13.0 (Stata Corporation, College Station, TX, USA) was used for descriptive and multivariable modelling. Latent class analyses were carried out in MPlus (Version 8.4, Muthen & Muthen) and maps created in ArcMap version 10.1 (ESRI, Redlands, CA).

The number, gender ratio and age distribution of cases and controls was described, with breakdown by season. As controls were frequency matched to cases by age, gender and calendar time, they were not representative of the underlying age and gender distribution of the source population. The 2011 census data was used to calculate the expected age and gender distribution in the general population in Zambezi Region, and define a second set of 641 hypothetical controls. These were compared to the age and gender distribution of cases using Pearson’s chi-squared test for association and used to calculate unadjusted odds ratios and corresponding 95% confidence intervals for these risk factors. Bivariate associations between all other exposures and case status were assessed using logistic regression with the Huber White sandwich estimator for standard errors, including a fixed effect to capture clustering at the health facility level and adjusting for age, gender, calendar quarter and year.

Collinearity between behavioral and environmental exposures was assessed during the model building process by comparing p-values within bivariate models and calculating variance inflation factors (VIF), where a VIF over 10 was taken to indicate collinearity. Reported frequency of sleeping under a net and sleeping under a net the previous night were strongly associated (p<0.0001), therefore the latter measure was retained in the model as a more reliable measure. Continuous variables were initially plotted using the ‘lowess’ command in STATA and divided into categories based on natural breaks or three to four centiles for the unadjusted analysis. All continuous variables were then assessed as either continuous or categorical in the final model and the best fitting version retained based on the Bayesian information criterion (BIC) value.

Multivariable logistic regression models were built using a backwards stepwise approach, retaining variables in the final model that had a p-value <0.05 or changed any adjusted odds ratio (aOR) more than 10%. Travel, agricultural work and net use were identified as time-varying exposures in the raw data, and plausible effect modification assessed a by adding covariate-by-time period interaction terms to the final multivariable model. In addition, a sensitivity analyses was run to investigate whether the results were affected by the period during which a disproportionate number of controls were recruited relative to cases (10 cases and 142 controls recruited outside of the main malaria transmission season of November to May). An interaction term was also evaluated to assess whether the risk associated with outdoor activities varied according to reported use of bite prevention measures and season. Residuals from the final model were exported and tested using Moran’s I for evidence of spatial autocorrelation in R.

A latent class analysis (LCA) was conducted to explore overlap in behavioral and epidemiological risk factors and to profile distinct high risk populations who may require specific intervention approaches. LCA is a statistical tool to identify homogenous, mutually exclusive groups or classes within a heterogenous population [35]. The LCA model included all variables indicating risk behaviors related to malaria in the univariate analysis, sex, and age group. All categorical variables were coded as dummy variables in MPlus. Multiple models were estimated with varying numbers of classes (from one to eight classes) and no covariates in MPlus, using the maximum likelihood solution from running 100 iterations for each model from randomly generated seed values. The Bayesian information criterion (BIC) was used to determine the best-fitting model, and checked against the adjusted BIC (aBIC), in the context of entropy (a summary measure of classification certainty) and epidemiological meaningfulness of class structure. For subsequent descriptive analyses, participants were allocated to the latent class for which they had the highest posterior membership probability.

### Ethical approval

Institutional Review Board approval for this study was obtained from the University of California San Francisco, the University of Namibia, and the Ministry of Health and Social Services of Namibia. Written consent by signature or finger print was provided from all consenting participants. Consent for individuals aged under 18 years was provided by a parent or guardian. Assent was collected for all children over 12 years old. All individuals identified as cases with positive RDT result received the national first-line malaria treatment (Coartem [artemether-lumefantrine]) in accordance with the existing standard of care at the health facility.

## Results

### Descriptive analysis

**Fig 2:**
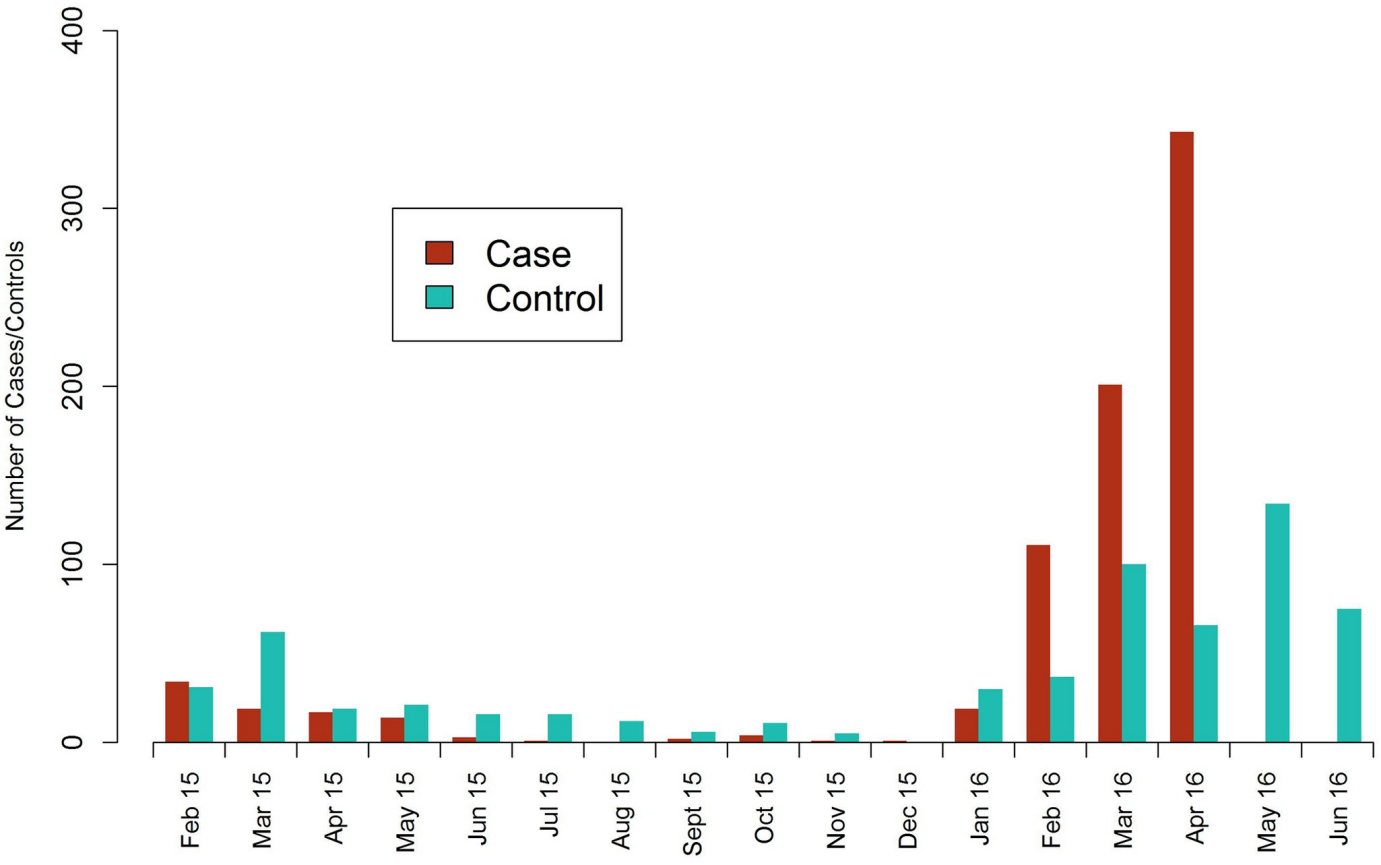
Monthly recruitment of 770 cases and 641 controls at six participating health facilities in Zambezi Region, Namibia between February 2015 and June 2016.

A total of 1417 individuals were tested by RDT and eligible to participate in the study, including 772 RDT+ malaria cases and 645 RDT- febrile controls. This includes 86% of all incident cases reported through routine surveillance systems in the period between January and April 2016 from the 6 included health facilities. Most cases who declined to participate did not provide a reason, but of six individuals who cited reasons: one case felt too ill while the others cited concerns around the procedures or lacked time to participate. A total of 770 cases and 641 controls were interviewed at the clinics, the majority of whom (87% of cases and 69% of controls) were recruited during the second transmission season in 2016 (Fig 2). This period coincided with a malaria outbreak across northern Namibia and other parts of Southern Africa [2].

Summary characteristics of malaria cases and controls are described for each of the two transmission seasons in Table 1. Control recruitment in a 2:1 ratio to cases was not possible in the second transmission season, due to the high burden of cases. The vast majority (99.5%) of participants reported they were residents within Namibia and most cases and controls had Namibian citizenship (83.3% and 90.6%), primary education or lower (78.8% and 71.5%) and reported relatively low net usage (39.4% and 58.8%) and spray coverage (33.4% and 40.3%) in their sleeping structure. Peak transmission in 2016 shifted to later in the season, with four-fifths (80.2%) of cases arising after February, compared to roughly two- thirds (64.8%) in 2015.

**Table 1:** Socio-demographics and select characteristics of 770 cases and 641 controls in Zambezi Region recruited between February 2015 and April 2016.

Characteristic	Season 1		Season 2		Total	
	Case (N = 96)	Control (N = 199)	Case (N = 674)	Control (N = 442)	Case (N = 770)	Control (N = 641)
Age (years)						
< 5	12 (12.8)	26 (13.4)	45 (6.7)	61 (13.6)	57 (7.4)	87 (13.6)
5–14	28 (29.8)	57 (29.4)	250 (37.0)	107 (23.9)	278 (36.1)	164 (25.6)
15–59	53 (56.4)	104 (53.6)	332 (49.1)	250 (55.9)	385 (50.0)	354 (55.2)
60 +	1 (1.1)	7 (3.6)	49 (7.2)	29 (6.5)	50 (6.5)	36 (5.6)
Male gender	51 (54.3)	102 (52.6)	366 (54.1)	201 (45.0)	417 (54.2)	303 (47.3)
Resides in Namibia	91 (96.8)	193 (99.5)	673 (99.6)	447 (100)	764 (99.2)	640 (99.8)
Citizenship						
Namibian	82 (87.2)	175 (90.2)	559 (82.7)	406 (90.8)	641 (83.3)	581 (90.6)
Foreign	12 (12.8)	19 (9.8)	117 (17.3)	41 (9.2)	129 (16.8)	60 (9.4)
Education						
None	22 (23.4)	58 (29.9)	186 (27.5)	129 (28.9)	208 (27.0)	187 (29.2)
Primary	59 (62.8)	92 (47.4)	340 (50.3)	179 (40.0)	399 (51.8)	271 (42.3)
Secondary	13 (13.8)	44 (22.7)	150 (22.2)	139 (31.1)	163 (21.2)	183 (28.6)
Slept under net	48 (51.1)	125 (64.4)	256 (37.9)	252 (56.4)	304 (39.5)	377 (58.8)
Sprayed ≤ 1 year	35 (37.2)	62 (32.0)	222 (32.8)	196 (43.8)	257 (33.4)	258 (40.3)
Don’t know	9 (9.6)	17 (8.8)	66 (9.8)	31 (6.9)	75 (9.7)	48 (7.5)
Migration^1^						
Not within past six months	82 (87.2)	176 (90.7)	611 (90.4)	418 (93.5)	693 (90.0)	594 (92.7)
Domestic migration	9 (9.6)	12 (6.2)	28 (4.1)	22 (4.9)	37 (4.8)	34 (5.3)
Cross-border migration	3 (3.2)	6 (3.1)	37 (5.5)	7 (1.6)	40 (5.2)	13 (2.0)
Recent mobility^2^						
None	82 (87.2)	157 (80.9)	607 (89.8)	367 (82.1)	689 (89.5)	524 (81.7)
Domestic (Low^3^ risk)						
Domestic (High risk)	11 (11.7)	34 (17.5)	54 (8.0)	77 (17.2)	65 (8.4)	111 (17.3)
Cross-border	1 (1.1)	3 (1.5)	15 (2.2)	3 (0.7)	16 (2.1)	6 (0.9)
Early in season	35 (36.8)	33 (16.8)	133 (19.7)	73 (16.3)	168 (21.8)	106 (16.4)

MMP: mobile and migrant population;

^1^ Within the past six months,

^2^ Within the past 30 days,

^3^ Non-endemic or lower endemic area (<10% of case burden in Zambezi Region).

#### Mobility patterns

A total of 198 participants (14%) reported taking at least one trip in the past 30 days, with a minority of cases and controls (1.2% and 2.5%) reporting more than one trip. Travel characteristics are reported in Table 2. Recent cross border travel was rare, but more common amongst cases than controls (2.2% and 1.1%). A majority of cross border travel was by Zambian citizens (68%) and a key location was nearby districts in Western Province, Zambia. In contrast, overnight travel within Namibia was less common amongst cases than controls (7.5% and 16.2%). Most (82%) overnight trips were within Zambezi Region, although a minority of participants traveled to other areas within Namibia.

**Table 2:** Travel characteristics of cases and controls reporting overnight travel in prior 30 days^1^, from a sample of 770 cases and 641 controls in Zambezi Region.

Characteristic	Domestic (Low risk)		Domestic (High risk)		Cross-border	
	Cases N = 14	Controls N = 50	Cases N = 49	Controls N = 60	Cases N = 18	Controls N = 7
Male	8 (57.1)	20 (40.0)	33 (67.4)	25 (41.7)	11 (61.1)	7 (100.0)
Age category:						
<5	1 (7.1)	5 (10.0)	2 (4.1)	6 (10.0)	3 (16.7)	1 (14.3)
5–14	0 (0.0)	10 (20.0)	11 (22.5)	17 (28.3)	5 (27.8)	0 (0.0)
15–59	13 (92.9)	35 (70.0)	32 (65.3)	36 (60.0)	9 (50.0)	5 (71.4)
60 +	0 (0.0)	0 (0.0)	4 (8.2)	1 (1.7)	1 (5.6)	1 (14.3)
Foreign citizenship	2 (14.3)	3 (6.0)	8 (16.3)	6 (10.0)	14 (77.8)	3 (42.9)
Early season (Nov-Feb)	9 (21.4)	9 (18.0)	14 (28.6)	15 (25.0)	15 (83.4)	1 (14.3)
Frequency:						
> 1 per month	3 (21.4)	11 (22.9)	16 (35.6)	11 (18.6)	1 (5.6)	0 (0.0)
< 1 per month	4 (28.6)	23 (47.9)	16 (35.6)	26 (44.1)	7 (38.9)	4 (57.1)
≤ 1 per year	7 (50.0)	14 (29.2)	13 (28.9)	22 (37.3)	10 (55.6)	3 (42.9)
Duration:						
< 1 week	7 (50.0)	32 (64.0)	29 (59.2)	30 (50.0)	3 (16.7)	3 (42.9)
1–4 weeks	6 (42.9)	16 (32.0)	14 (28.6)	27 (45.0)	3 (16.7)	4 (57.1)
1 month +	1 (7.1)	2 (4.0)	6 (12.2)	3 (5.0)	12 (66.7)	0 (0.0)
Reason for travel:						
Occupation	2 (14.3)	8 (16.0)	9 (18.4)	11 (18.3)	2 (11.1)	0 (0.0)
Education	0 (0.0)	2 (4.0)	6 (12.2)	3 (5.0)	0 (0.0)	0 (0.0)
Family	10 (71.4)	32 (64.0)	31 (63.3)	39 (65.0)	16 (88.9)	6 (85.7)
Other^2^	2 (14.3)	8 (16.0)	3 (6.1)	7 (11.7)	0 (0.0)	1 (14.3)
Use of bite prevention	3 (21.4)	7 (14.0)	5 (10.2)	10 (16.7)	3 (16.7)	3 (42.9)
Destination:						
northern Namibia (Zambezi)	13 (92.9)	47 (94.0)	49 (100.0)	55 (91.7)	-	-
northern Namibia (Other)	1 (7.1)	0 (0.0)	0 (0.0)	5 (8.3)	-	-
Other Namibia (Hardap, Khomas)	0 (0.0)	3 (6.0)	-	-	-	-
Western Province, Zambia	-	-	-	-	15 (83.3)	4 (57.1)
Other, Zambia	-	-	-	-	1 (5.6)	3 (42.9)
Cuando Cubango, Angola	-	-	-	-	2 (11.1)	0 (0.0)

^1^ If >1 destination, characteristics summarized for destination with highest risk,

^2^ Funerals, health, church and shopping.

**Fig 3:**
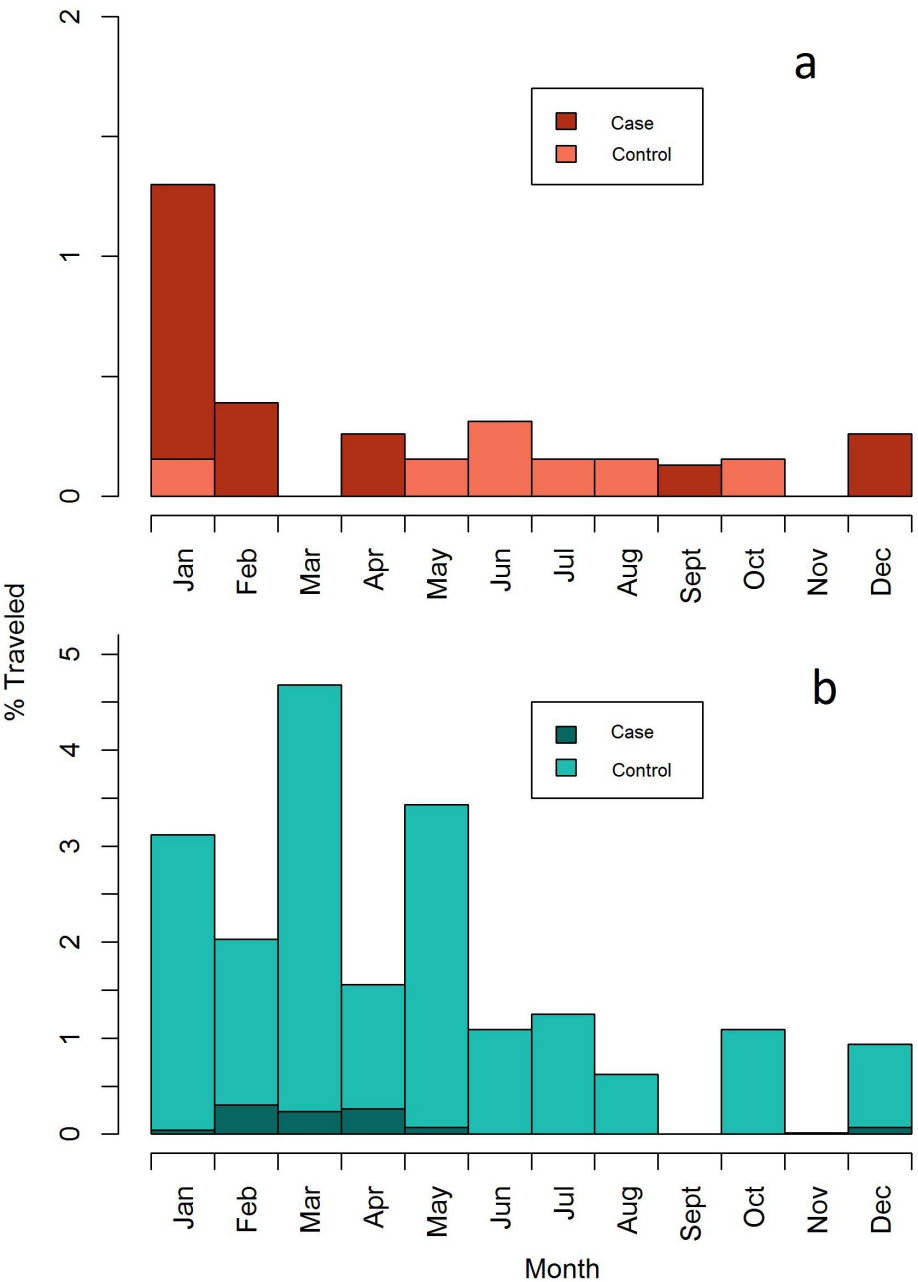
Relative frequency histogram presenting the proportion of cases and controls reporting cross border (a) and domestic (b) travel by month.

Both domestic and cross-border travel tended to be seasonal and occurred during the rainy season (Fig 3). However, the majority of cross-border travel in cases (82%) occurred early in the transmission season compared to in controls (82% vs 14%) (Table 2), indicating a high-risk window of travel time December to February that corresponds with the agricultural season and holiday festivities.

### Risk factors for malaria

**Fig 4:**
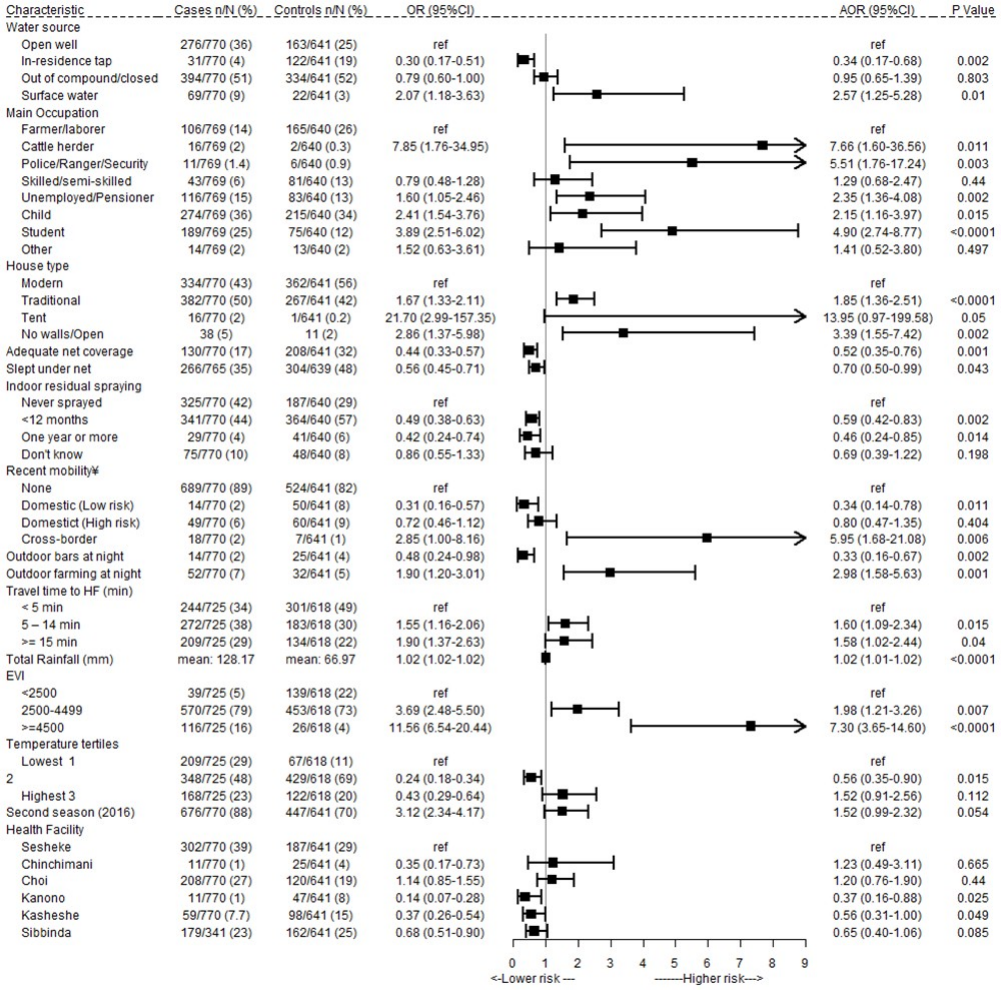
Unadjusted and adjusted odds ratios for selected exposures associated with the risk of symptomatic malaria in Zambezi Region, adjusting for matching variables (age, gender, calendar quarter).

A full table of unadjusted odds ratios for potential risk factors is included in S2 Table. Variables retained in the final model are described below and unadjusted and adjusted odds ratios for these selected variables are shown in Fig 4. After adjusting for all risk factors, there was no evidence supporting spatial correlation in the residuals (Moran’s I p = .706).

#### Socio-demographic risk factors

Using the census controls as a reference, the crude odds of malaria was higher in males (Odds ratio (OR) 1.25 95% CI 1.00–1.54; p = 0.04) and lower in children aged 0–4 years, compared to older age categories. The odds of malaria were 0.63 (95% CI 0.45–0.88) in the youngest age group and ranged from 1.09 (95% CI 0.74–1.26) in adults aged 15–59 years to 1.74 in children aged 5–14 years (95% CI 1.43–2.11).

Non-Namibian nationality was identified as a risk factor in the unadjusted analysis of the study data (S2 Table), as its effects were accounted for by associated variables (cross-border travel, migration and occupational categories). Use of an in-residence tap for drinking water was protective compared to an open well (Adjusted Odds Ratio (aOR): 0.34 95%CI 0.17–0.69), while cases were more likely to report surface water as their main source of drinking water (aOR: 2.10 95%CI 1.03–4.27). Other socioeconomic indicators, such as lower education and a lower score on the PCA asset index, were associated with a higher risk of malaria in the unadjusted but not adjusted analysis (S2 Table).

#### Occupation and outdoor activities

The risk of malaria was higher in cattle herders (aOR: 4.46 95%CI 1.05–18.96), members of the police or other security personnel (aOR: 4.60 95%CI: 1.16–18.16), and pensioners/unemployed persons (aOR: 2.25 95%CI 1.24–4.08) compared to agricultural workers (most common). After adjusting for age, children (aOR 2.28 95%CI 1.13–4.59) and students were at higher risk of malaria (aOR 4.32 95%CI 2.31–8.10). There were high levels of overlap between specific occupational categories and reported outdoor behaviors associated with malaria in the unadjusted analyses (S2 Table), such as playing and studying outside at night. Collinear behavioral variables were dropped from the final model but retained in the latent class analysis to better profile sub-groups with similar characteristics.

In the final model (Fig 4), agricultural work carried out between sundown and sunup was independently associated with a higher risk of malaria (aOR: 2.09 95%CI 1.12–3.87), whereas frequenting outdoor bars at night was associated with four-fold reduction in the odds of malaria (aOR: 0.38 95%CI 0.17–0.85). While there was insufficient power to test interactions in the final model, stratified chi-squared tests and a bivariate model of gender and bar attendance suggested that this protective effect may be present in females (*χ*^2^ p = 0.005 and OR: 0.10, 95%CI [0.01–0.87], p = 0.04) but not males (*χ*^2^ p = 0.21 and OR: 0.71, 95%CI [0.31–1.61], p = 0.41). There was insufficient power to assess whether the risk associated with outdoor activities varied with use of bite prevention, with a minority of cases (3.6%) and controls (6.7%) reporting using any form of protection against mosquito bites while engaged in outdoor activities between sunup and sundown.

#### Travel-related risk factors

Overnight cross border travel within the past month was associated with an increased risk of malaria (aOR: 10.55 95%CI 2.94–37.84), while domestic travel to low risk destinations was protective (aOR: 0.38 95%CI 0.17–0.85).

#### Housing and malaria interventions

Traditional home construction and sleeping in an open structure was associated with an increased odds of malaria (aOR: 2.01 95%CI 1.45–2.79 and aOR: 4.76 95%CI: 2.14–10.57) compared to homes built using modern construction materials. Cases were ten times more likely to sleep in a tent than controls, but there was weak evidence to support this association in the multivariable model (aOR: 16.34 95%CI 0.28–957). Openings to the outside (such as a gap between the walls and roof) were present in all traditional structures and so left out of the final model.

All malaria preventive activities were strongly protective against malaria. The risk of malaria was lower in homes with adequate household net coverage and in persons who reported sleeping under a net the previous night (aOR: 0.63 95%CI 0.42–0.94 and aOR: 0.61 95%CI 0.42–0.87), suggesting a household level protective effect persists after accounting for individual net use. An interaction between net use and occupational category suggested that individual net use was protective in children, unemployed persons and skilled/semi-skilled workers (p = 0.006, 0.099, and 0.015), but not in students, agricultural workers, or police/security guards (p = 0.465, 0.997, and 0.137). Neither of the two controls that were occupied as cattle herders reported using a net and so this interaction was not included in the final model. Any indoor residual spraying on the walls of the sleeping structure was associated with a lower risk of malaria, either within the past year (aOR: 0.63 95%CI 0.44–0.90) or more than one year ago (aOR: 0.44 95%CI 0.23–0.82) compared to never having been sprayed.

#### Environmental and seasonal risk factors

Residing more than 5 minutes from a health facility was associated with a higher risk of malaria (aOR: 1.63 95%CI 1.10–1.67). Environmental factors were strongly associated with malaria risk, with each millimeter of rainfall in the month prior to presentation associated with a 2% increase in the adjusted odds of malaria (aOR: 1.01 95%CI: 1.01–1.02). Similarly, higher levels of vegetation measured by EVI were associated with increasing odds of malaria (Fig 4). After adjusting for other environmental risk factors, higher levels of LST remained associated with lower risk of malaria.

Increased levels of rainfall and vegetation in 2016 compared to 2015 (p<0.0001 by t-test) likely partly account for higher levels of malaria transmission. However, even after accounting for environmental variables in the final model and variation between calendar quarters, there was weak evidence of higher risk of malaria in 2016 (aOR: 1.60 95%CI 1.02–2.52).

#### Sensitivity analyses

Further analyses were performed in which the characteristics of controls recruited during the main transmission season (between 1 November and 31 May) were compared to those recruited outside the main transmission season; no notable changes were evident in the risk factors for malaria using the full dataset compared to the restricted dataset but estimates were slightly more precise (data not shown).

### LCA results: High risk profiles

**Fig 5:**
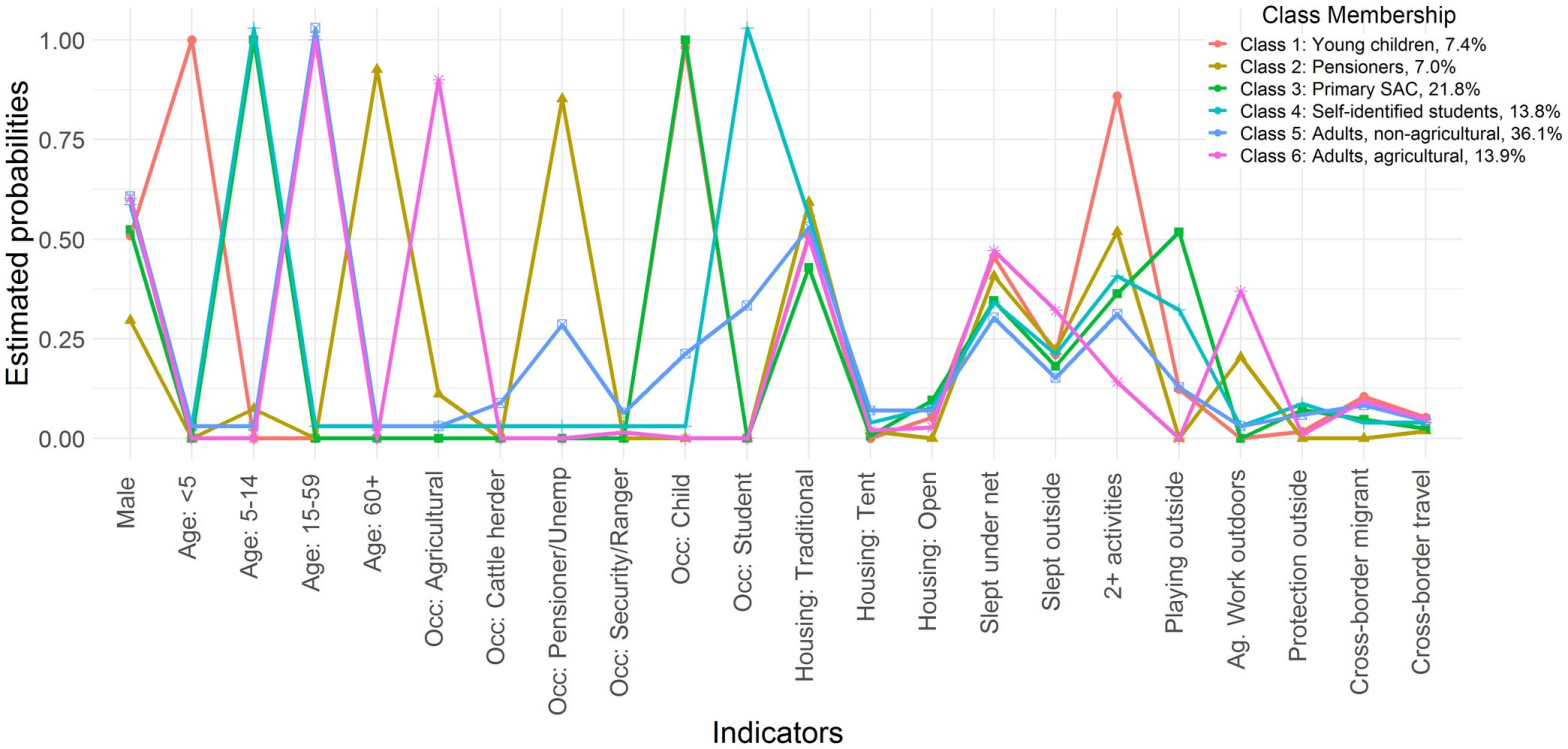
Profile plot of estimated indicator probabilities and latent class proportions for all six latent classes of malaria risk in a sample of malaria cases from Zambezi Region. The item-response probabilities represent the probability of a positive response on a variable, given membership in a given class.

After comparison of fit statistics, a model with six classes was found to be the best fit for the data [BIC (6 class) = 11343; BIC (5 class) = 11423]; higher numbers of classes resulted in numerical difficulties. The conditional probabilities of each malaria risk factor associated with clusters are shown in Fig 5 and S3 Table, and group characteristics in Table 3. Clusters were named to best represent the characteristics of each cluster, which were strongly correlated with occupation, age and select behavioral risk factors. Use of malaria prevention was similar across classes, suggesting low net use and IRS are likely to contribute to transmission in all groups.

**Table 3:** Profiles of six high risk malaria clusters based on individual risk factors, including select class conditional probabilities (full in S3 Table) and key socio-demographic and behavioral indicators.

Characteristic	Class 1 Young children	Class 2 Namibian pensioners	Class 3 SAC	Class 4 Students	Class 5 Outdoor exposures	Class 6 Adult agr. workers
Number (%)	57 (7%)	54 (7%)	168 (22%)	106 (14%)	278 (36%)	107 (14%)
Median age [IQR](years)	3 [2–4]	69 [62–76]	8 [7–11]	11 [9–13]	21 [17–30]	33 [25–46]
Male	51%	30%	52%	56%	58%	60%
Primary occupation(s)	Child (100%)	Pensioner (72%) Unemployed (13%) Agr./fishing (11%)	Child (100%)	Student (100%)	Unemployed (25%) Cattle herder (6%) Student (30%) Police/security (4%)	Agr./fishing (93%) Sales/service (4%)
Foreign citizenship	28%	6%	12%	13%	15%	31%
Migrant:						
Domestic	5%	2%	0%	3%	9%	4%
Cross-border	11%	0%	5%	1%	5%	9%
Recent travel:						
Domestic	5%	7%	2%	7%	12%	12%
Cross border	5%	2%	2%	1%	1%	5%
Lowest SES asset tertile	70%	56%	53%	58%	51%	41%
Housing:						
Modern	44%	39%	47%	42%	42%	45%
Traditional	51%	59%	43%	53%	50%	50%
Tent	0%	2%	1%	1%	4%	2%
Open	5%	0%	9%	5%	4%	3%
Never sprayed	49%	44%	45%	34%	42%	41%
Net use prior night	46%	40%	35%	31%	28%	47%
Median # activities	3	3	2	2	2	1
Time of outdoor activities						
After sunset	14%	26%	59%	62%	61%	52%
Late evening/all night	0%	4%	1%	0%	3%	4%
Early morning	2%	30%	17%	21%	35%	64%
Agricultural work at night	0%	20%	0%	0%	0%	38%
Sleep outdoors at night	21%	22%	18%	18%	13%	32%
Playing outdoors at night	12%	0%	52%	29%	10%	0%
>15min from health facility	26%	17%	30%	37%	30%	22%
Health facility						
Sesheke	32%	46%	43%	19%	44%	40%
Choi	26%	22%	30%	28%	26%	27%
Kasheshe	7%	6%	11%	10%	7%	2%
Sibbinda	32%	21%	11%	41%	21%	28%
Kanono	2%	2%	1%	1%	2%	2%
Chinchimani	2%	4%	2%	1%	1%	1%
Early in season	21%	11%	30%	12%	19%	30%
2016 season	79%	94%	90%	91%	88%	82%

IQR: inter-quartile range; SES: socioeconomic status.

*Class 1 –Young children (n = 57*, *7*.*4%)*: were a smaller risk group but included a relatively high proportion of migrants (15%) and cross-border travelers (5%). The group had the highest proportion of foreign-born individuals (28%) and were largely rural and poor, with the majority living further than 5 minutes from a health facility (68%) and in the lowest socioeconomic tertile (70%). This group was less likely than other classes to have been active outdoors between sunset and sunrise, suggesting that transmission occurs in the home or while sleeping outdoors.

*Class 2 –Namibian pensioners (n = 54*, *7*.*0%)*: were another small risk group comprised of individuals identified as pensioners or unemployed who were predominantly Namibian (92%), local residents (98%), female (70%) and aged 60 years and above. A fifth (20%) reported agricultural work at night with most outdoor activities reported after sunset and early in the morning. These cases predominately arose later in the transmission season (89%) and during the 2016 outbreak (94%).

*Class 3 –Primary school-age children (n = 168*, *21*.*8%)*: were a large risk group and included children aged 5–14 years (median age 8 and IQR 8–11 years). Members of this group were more likely to sleep in the open (9%) and without a net the previous night (65%). Reported outdoor exposure was primarily after sunset, and members engaged in play or domestic activities. These cases were identified earlier in the transmission season (25% in February) compared to many other groups and had a relatively large burden in the 2016 outbreak, along with pensioners and students.

*Class 4 –(Self-identified) Students (n = 106*, *13*.*8%)*: were at higher risk of malaria than those who self-identified as children, for largely unknown reasons. These individuals tended to be slightly older (median age 11 and IQR = 9–13 years) than Class 3. Compared to other groups, members of this class were more likely to be male and live more than 15 minutes from a health facility (37%). Although IRS at their main place of residence was relatively high, the study did not capture sleeping at a school dormitory and many individuals reported sleeping without a net the previous night (69%).

*Class 5 –Adults; non-agricultural workers (n = 278*, *36*.*1%)*: broadly included young adults (median age 21 and IQR 17–30 years) with outdoor non-agricultural occupations, such as cattle herders, security personnel and police officers, and rangers. This class also included a subset of younger unemployed persons and older students who were included here as a function of age and lack of agricultural exposures. While overall 15% of individuals in this class identified themselves as non-Namibian, there was variation within occupational subgroups. For example, a higher proportion of cattle herders were Zambian (69%) and were diagnosed early in the transmission season (31%) compared to unemployed persons (16% Zambian and 10% early in the season). This class had the lowest net use of all groups (28%).

*Class 6 –Adults; agricultural workers (n = 107*, *13*.*9%)*: included adults (median age 33 and IQR 25–46 years) who identified as agricultural workers. While this broad occupational category did not have a high risk of malaria, other high risk characteristics tend to co-occur and define members of this group, including agricultural work at night (38%), foreign citizenship (31%), cross-border migration (9%) and recent domestic or cross-border travel (12% and 5%). These cases were identified earlier in the transmission season (28% in February) compared to many other groups.

## Discussion

In this assessment of risk factors associated with symptomatic malaria in Zambezi Region, our findings support the presence of 6 distinct malaria high-risk subgroups primarily defined by key risk factors like occupation and age. In terms of the burden of disease, key groups were adults with outdoor occupational exposures in Classes 5 and 6 (including subpopulations of cattle herders, unemployed persons, and farmers who work outdoors at night), and school age children. Use of malaria prevention was similarly low within these groups, suggesting that they are frequently missed by intervention campaigns and some sleep in informal or temporary housing structures without nets or protected by IRS. Environmental factors were strongly related to malaria risk, and occupational risk factors indicate that outdoor biting and transmission away from the home is likely to be important in this setting. Based on these findings, tailored strategies are warranted to address transmission within different subpopulations and improve overall coverage and impact of public health interventions. This study describes a robust methodology for understanding populations at greater risk of malaria transmission where risk factors overlap or co-occur.

Overall, study findings point towards exposures occurring outdoors and away from home, and a need to investigate worksites as potentially important sites of transmission linking villages within foci. Results from our analyses suggest that adults with outdoor occupational exposures, including field workers and cattle herders working in agricultural areas, are an important risk group in this setting. A cross-sectional survey in 2015 in Zambezi Region reported a similar five-fold increase in the odds of malaria infection associated with agricultural occupations and cattle herders, detected by loop-mediated isothermal amplification [30]. Despite the ubiquity of malaria in rural, farming areas across sub-Saharan Africa and well documented risk in seasonal migrants and permanent agricultural workers in Ethiopia [36, 37], few studies have explicitly investigated how agricultural exposures (including occupation and land cover) influence individual malaria risk [38, 39]. The effect of agricultural practices and crop types may vary, with differences in effect estimates influenced by vector bionomics, human population susceptibility and other mediating factors like access to and use of malaria prevention [40–42]. Recent entomological assessments within the study area found that vectors will bite outdoors throughout the hours of 7pm to 7am, with peaks between 10-12pm and 3-5am [43]. Amongst agricultural workers in the current study, the marginal effect of working outdoors early in the morning and/or evenings translated to a 12% increase in the odds of being a case, reinforcing the importance of behavioral differences as a mediator of vector-human contact. Other outdoor occupations such as cattle herders, security guards, police offices and rangers had a relatively high risk of malaria, and unemployed persons also reported frequent outdoor exposures such as agricultural activities and sleeping outside at night. Surprisingly, after controlling for other risk-factors in the model, spending time outdoors at bars between biting hours was associated with a lower risk of malaria [44]. This protective effect was observed only in females, some of whom worked at the location, and suggests the presence of uncontrolled confounding or selection bias in relation to behavioral risk or treatment seeking behaviors. Alternatively, this finding could be due to chance, given the low prevalence of this exposure and small sample size.

The study found a higher risk of malaria in cross-border travelers compared to those who did not travel or those who traveled domestically, despite the low occurrence of cross-border travel. Trips were overwhelmingly for family and work-related reasons, with cross border trips of longer duration and lower frequency than domestic travel. Specific sub-groups of cases, including cattle herders, agricultural workers and young children, were more likely to identify as non-Namibian (70%, 31% and 28% respectively) and migrants or cross-border travelers compared to other groups. Cross-border travelers are a known high risk group across southern Africa, and their vulnerability to infection is attributed to lower access to health services and use of malaria prevention at their destination, in combination with travel to/from higher transmission settings [4, 45–48]. Domestic travel to low risk areas (defined as areas with no transmission or <10% of the regional total in Zambezi Region) was protective against malaria and there was no evidence of an association between malaria and travel to higher risk areas within Namibia. Research by Tessema et al. complements these findings and, using genetic data derived in part from this study, demonstrated high levels of genetic parasite connectivity to neighboring countries and in particular, Western Zambia [49]. In this setting, it is likely that health facility data underrepresent seasonal workers and young children who may accompany their parents for seasonal work, due to the remoteness of worksites and potential concerns around payment in relation to residency status. This study highlights the diversity of mobile populations in northern Namibia, and illustrates the need to tailor community-based solutions to local contexts to address population mobility [50].

Self-identified students accounted for nearly a quarter of malaria cases and school-aged children represented half of all cases in the study area. Malaria is prevalent in school-aged populations across moderate and high transmission contexts [51–53], behavioral risk factors that lead to increased exposure of these age groups to malaria as well as effective control measures that target younger children. In this study, there was a clear shift in the burden of cases towards younger age groups between 2015 and the outbreak in 2016 (Table 1), reflecting the impact of the epidemic on a susceptible population in a normally low endemic context. Net and IRS coverage was insufficient across all populations, but learners in grades 9–12 may be particularly exposed when staying away from home during exam periods (February to November). During this time, students typically stay on or near school grounds and sleep in tents or hostels with little or no access to bednets. School-based malaria interventions are a staple intervention in higher burden settings and in Namibia, offer a targeted approach to improve intervention coverage and hence impact on transmission dynamics. School-based interventions are able to leverage a trained cadre of teachers, making them cost-efficient agents for combining malaria prevention education with distribution of LLINs [54, 55] and have been successfully integrated with existing neglected tropical disease (NTD) mass drug administration campaigns [56]. Housing improvements at dormitories would be a low cost and potentially high impact intervention. Preventive treatment (PT) of malaria in school-age children has shown impact on prevalence of asexual parasites and gametocytes, clinical malaria and anaemia across transmission settings [57, 58]. Vector control and rapid access to case management through school platforms would be relatively cost-effective, while highly targeted intermittent PT to hotspots might be feasible, in conjunction with drug resistance monitoring and management [59].

Our study supported many established risk factors for malaria including: housing type, malaria prevention measures, including individual net use, household net coverage, and IRS coverage, and environmental factors including weather and distance to health facility. A higher risk of malaria was associated with traditional or informal housing structures, including those with open eves, open sides and tents. These findings are corroborated by the literature, in which improved housing is consistently associated with reduced risk of a range of malaria outcomes [29, 60–63] and provides barriers to mosquito entry [64, 65]. The protective effect of ITNs is well established in areas of stable and unstable malaria transmission [66], and a universal net distribution took place in Zambezi region in 2013 [1]. Since this, their distribution has been restricted to vulnerable populations through antenatal clinics and, more recently, in a targeted approach to some of the high-risk populations identified in this study. In elimination settings, programs may be challenged by lower uptake of some interventions due to the perception that malaria is no longer a risk, which should be managed through targeted messaging and health promotion [67]. In contrast, IRS is currently a cornerstone of the malaria elimination strategy in Namibia, which used predominantly DDT for malaria vector control up till 2017. DDT is typically expected to be effective over a six to 12 month time frame [68], but this study found a protective effect of IRS within the past year or ever, compared to never having been sprayed. Some of this apparent effect may be capturing residual confounding related to socioeconomic status or proximity to a road/village center. Taken within the context of low coverage of malaria preventive measures, these findings suggest that a large proportion of transmission still occurs indoors in Zambezi region and effective control will require targeted scale up of these interventions. Climatic risk factors including temperature, vegetation (EVI) (a proxy measure of relative humidity [69]) and precipitation in the month prior to diagnosis suggest that transmission varies on relatively small scales [4, 70, 71]. After accounting for rainfall, increased risk associated with higher EVI and lower temperatures is likely to relate to greater availability of larval habitats [72] and lower thermal optima for mosquito and parasite development [73]. These relationships are difficult to disentangle from seasonal variation in risk-behaviors, such as agricultural work, which occur in areas with higher vegetation and during the wet season. Increased distance to health facility was associated with a higher risk of malaria, which likely reflects lower access to care and socioeconomic vulnerability, as found in previous studies using DHS data [28]. Individuals who reported surface water as their main source of drinking water were more likely to have malaria, and may indicate the presence of vector breeding sites near a household or act as a proxy measure of socioeconomic status.

The main limitation of this study lies in its health facility-based design, which limits inference to symptomatic and higher density infections diagnosed by RDT and treatment seeking populations. Case recruitment at health facilities remains the most feasible option in low endemic settings, and selection of controls from this same population avoids documented selection biases due to treatment seeking [4, 28]. However, sub-populations with lower treatment seeking behaviors are likely to be underrepresented in both case and control data. In addition, the large malaria outbreak across southern Africa in 2016 presented a key operational challenge during implementation, and resulted in a higher case burden, challenges in recruitment of controls and altered transmission dynamics. Associations and identified risk factors in non-epidemic years may differ. In general, matching was successful only in the first transmission season, when incident case numbers were lower and it was possible to recruit control profiles within a shorter time frame. The results from the sensitivity analysis, which restricted the sample to controls recruited within the same timeframe as incident cases, did not result in changes to the model. Potential sources of measurement error that could introduce misclassification in exposures include recall bias for self-reported measures of net usage and travel history, geolocation errors and uncertainty around relevant periods of exposure due to variation in the length of time between infection and diagnosis. Finally, statistical power to assess interactions was limited by the small sample size of some occupational sub-groups and low prevalence of other specific risk factors.

The findings from this study have important programmatic implications. Routine surveillance data should collect detailed occupational data to allow monitoring within sub-groups and IRS should be scaled up to achieve high coverage of vector control interventions amongst high risk groups. Intervention approaches can be targeted and tailored for specific groups and delivery strategies adapted to leverage existing occupational and educational networks and thereby improve access and community engagement in target populations. Community and school-based intervention campaigns are a natural fit as community leaders and teachers are likely to know who is travelling or engaged in particular occupational categories. Ethiopia implemented a successful community-based test and treat strategy in the home villages of returning migrant workers [74]. Police and security services are another easy group to target through intersectoral engagement and existing organizational structures, which has been done successfully in the Greater Mekong Subregion [75]. Worksites where seasonal agricultural workers congregate away from their home and experience gaps in protection are another access point for targeted interventions. Existing quantitative and qualitative methods used in HIV can provide effective tools to adapt and integrate routine surveillance and response activities for high-risk populations [76]. Furthermore, there is a need to pair entomological data collection with targeted surveys to understand the role of vector characteristics, including seasonal patterns and biting behaviors, on transmission at these locations. Future work is needed to understand the role of malaria high risk populations in driving transmission and the potential impact of targeted interventions.

## Conclusions

In this low endemic, cross-border setting, there are distinct malaria high risk populations with clearly identified exposures to infection and gaps in preventative interventions. Targeted and tailored intervention strategies are needed to extend coverage to these risk groups, including delivery through schools and worksites. Low IRS coverage and ITN use, coupled with outdoor exposures, suggest that targeted scale up of vector control activities should also consider specific interventions to address outdoor transmission and provide protection against outdoor biting. Adapting routine malaria surveillance and response to include high-risk populations is an area of growing concern as countries move closer to elimination and seek to prevent outbreaks and reintroduction of parasites through cross border movement from neighboring endemic areas. The findings from this study and other surveillance data are increasingly used to guide detailed formative work to support programs in planning for targeted surveillance and response strategies.

## Supporting information

S1 Table Potential environmental covariates. (PDF)

S2 Table Number, percent and univariate analysis for all measured exposures. (PDF)

S3 Table Latent class analyses among malaria cases: Probability of latent class membership and item-response probabilities within each of the six clusters. (PDF)
