# Supplementary material for: Malaria risk factors in northern Namibia: The importance of occupation, age and mobility in characterizing high-risk populations
Source: PLoS One. 2021 Jun 25;16(6):e0252690. doi: 10.1371/journal.pone.0252690 (PMC8232432; doi:10.1371/journal.pone.0252690)
Supplement: S1 Table — (PDF) [file pone.0252690.s001.pdf]

S1 Table. Potential environmental covariates

Covariate	Description	Units/scale	Spatial resolution	Temporal resolution	Source
Precipitation	Total precipitation in	Millimeters (mm)	~5 km	Monthly	Climate Hazards Group InfraRed Precipitation with Station (CHIRPS)
EVI	Enhanced Vegetation Index	-	1 km	8 day composites averaged for month	Moderate Resolution Imaging Spectroradiometer (MODIS)
Temperature (day)	Mean land surface temperature (day)	Degrees Celsius	1 km	8 day composites averaged for month	Moderate Resolution Imaging Spectroradiometer (MODIS)
Elevation	Average height above sea level	Meters	90 m	-	Shuttle Radar Topography Mission [http://srtm.usgs.gov/]
Distance to nearest health facility	Euclidean distance to road	Kilometers	1 km	-	Derived using R and health facility shapefile
Distance to border	Euclidean distance to border	Kilometers	1 km	-	Derived using R and country shapefile
