# Supplementary material for: Malaria risk factors in northern Namibia: The importance of occupation, age and mobility in characterizing high-risk populations
Source: PLoS One. 2021 Jun 25;16(6):e0252690. doi: 10.1371/journal.pone.0252690 (PMC8232432; doi:10.1371/journal.pone.0252690)
Supplement: S2 Table — (PDF) [file pone.0252690.s002.pdf]

S2 Table. Number, percent and univariate analysis for all measured exposures							
	**Cases**		**Controls**		**Unadjusted[1] Odds**		
**Characteristic**	**N**	**(%)**	**N**	**(%)**	**OR**	**95% CI**	**P-value**
**Socio-demographics**							
Foreign citizenship	129	16.8	60	9.4	2.25	(1.6-3.2)	**<0.0001**
Education level							
Secondary & above	163	21.2	183	28.6	1	-	-
Primary	399	51.8	271	42.3	1.64	(1.2-2.3)	**0.003**
None	208	27.0	186	29.1	1.47	(1.0-2.1)	**0.040**
Water source							
Open well	276	35.8	163	25.4	1	-	-
In-residence tap	31	4.0	122	19.0	0.30	(0.2-0.5)	**<0.0001**
Out of compound/closed	394	51.2	334	52.1	0.79	(0.6-1.0)	**0.096**
Surface	69	9.0	22	3.4	2.07	(1.2-3.6)	**0.011**
Toilet type							
None/open	754	97.9	613	95.6	1	-	-
Any latrine	16	2.1	28	4.4	0.61	(0.3-1.3)	0.181
Asset index (mean, sd)[2]	-0.29	0.05	0.35	0.07	0.81	(0.8-0.9)	**<0.0001**
Owns cattle	137	17.8	190	29.6	0.57	(0.4-0.8)	**<0.0001**
Main Occupation (2 missing)							
Agricultural	106	13.8	165	25.8	1	-	-
Cattle herder	16	2.1	2	0.3	7.84	(1.8-35.8)	**0.007**
Police/Ranger	7	0.9	4	0.6	2.43	(0.7-9.0)	0.182
Security guard	4	0.5	2	0.3	2.85	(0.6-13.6)	0.190
Skilled/semi-skilled	43	5.6	81	12.7	0.79	(0.5-1.3)	0.337
Unemployed/Pensioner	116	15.1	83	13.0	1.61	(1.1-2.5)	**0.030**
Child	274	35.6	215	33.6	2.41	(1.5-3.8)	**<0.0001**
Student	189	24.6	75	11.7	3.89	(2.5-6.0)	**<0.0001**
Other	14	1.8	13	2.0	1.52	(0.6-3.6)	0.352
**Housing**							
House type							
Modern	334	43.4	362	56.5	1	-	-
Traditional	382	49.6	267	41.7	1.67	(1.3-2.1)	**<0.0001**
Tent	16	2.1	1	0.2	21.7	(3.0-157)	**0.002**
Open/no walls	38	4.9	11	1.7	2.9	(1.4-6.0)	**0.005**
Open eaves	502	65.2	395	61.6	1.31	(1.0-1.7)	**0.028**
Sleeps near cattle	91	11.8	158	24.7	1.35	(0.8-2.1)	0.231
**Malaria prevention**							

Indoor residual spraying (1 missing)								
	Never sprayed	325	42.2	187	29.2	1	-	-
	< 12 months	341	44.3	364	56.9	0.49	(0.4-0.6)	**<0.0001**
	One year or more	29	3.8	41	6.4	0.42	(0.3-0.7)	**0.001**
	Don't know	75	9.7	48	7.5	0.86	(0.5-1.3)	0.496
Adequate net coverage[3]		130	16.9	209	32.6	0.44	(0.3-0.6)	**<0.0001**
Regular net use								
	Never	466	60.5	264	41.2	1	-	-
	Sometimes	61	7.9	85	13.3	0.56	(0.4-0.8)	**0.004**
	Always	243	31.6	292	45.6	0.47	(0.4-0.6)	**<0.0001**
Slept under net (7 missing)		266	34.8	304	47.6	0.56	(0.4-0.7)	**<0.0001**
**Outdoor activities between sunset and sunrise[4]**								
Number of outdoor activities								
	None	271	35.2	272	42.4	1	-	-
	1	328	42.6	268	41.8	1.08	(0.8-1.4)	0.554
	2+	171	22.2	101	15.8	1.53	(1.1-2.1)	**0.012**
Timing of outdoor activities								
	None	276	35.8	274	42.8	1	-	-
	After sunset	245	31.8	180	28.1	1.09	(0.8-1.5)	0.536
	Early morning	65	8.4	71	11.1	1.14	(0.8-1.8)	0.526
	Early morning and after sunset	169	22.0	105	16.4	1.29	(0.9-1.8)	0.135
	Late evening/all night	15	2.0	11	1.7	1.34	(0.6-3.1)	0.498
Frequency of (any) activities								
	None	288	37.4	288	44.9	1	-	-
	1-6 times per week	178	23.1	138	21.5	1.17	(0.9-1.6)	0.319
	7+ times per week	304	39.5	215	33.5	1.17	(0.9-1.5)	0.260
Work (farming)		718	93.3	609	95.0	1.90	(1.2-3.0)	**0.006**
Work (other)		738	95.8	615	95.9	1.10	(0.6-1.9)	0.753
Collecting water		609	79.1	533	83.2	1.20	(0.9-1.6)	0.239
Playing		618	80.3	576	89.9	1.55	(1.1-2.2)	**0.015**
Visiting bars		756	98.2	616	96.1	0.48	(0.2-1.0)	**0.043**
Studying		742	96.4	620	96.7	1.2	(0.6-2.3)	0.530
Weeding/fieldwork		737	95.7	614	95.8	1.0	(0.6-1.8)	0.908
Cow herding		760	98.7	636	99.2	1.21	(0.4-3.4)	0.718
Slept outside (4 missing)		142	18.5	80	12.5	1.77	(1.3-2.4)	**<0.0001**
Use of bite prevention		28	3.6	43	6.7	0.45	(0.3-0.8)	**0.003**
**Migration and recent travel**								
Migration[5]								
	Not within past six months	693	90.0	594	92.7	1	-	-

Domestic migration	37	4.8	34	5.3	1.10	(0.6-1.9)	0.750
Cross-border migration	40	5.2	13	2.0	3.36	(1.6-7.1)	**0.002**
Recent mobility[6]							
None	689	89.5	524	81.8	1	-	-
Domestic (Low risk)	14	1.8	50	7.8	0.31	(0.2-0.6)	**<0.0001**
Domestic (High risk)	49	6.4	60	9.4	0.72	(0.5-1.1)	**0.14**
Cross-border	18	2.3	7	1.1	2.85	(1.0-8.2)	**0.05**
Used bednet during trip	11	1.4	21	3.3	0.59	(0.3-1.3)	0.188
Trip duration							
No travel	689	89.5	524	81.8	1	-	-
Less than 1 week	39	5.1	66	10.3	0.61	(0.4-1.0)	**0.030**
1 – 4 weeks	23	3.0	46	7.2	0.46	(0.3-0.8)	**0.008**
1 month or more	19	2.5	5	0.8	2.91	(1.0-8.1)	**0.043**
**Environmental (68 missing)**							
Distance to nearest health facility							
< 5 min	244	33.7	301	48.7	1	-	-
5 – 14 min	272	37.5	183	29.6	1.54	(1.2-2.1)	**0.003**
≥ 15 min	209	28.8	134	21.7	1.90	(1.4-2.6)	**<0.0001**
Less than 10km to border	287	39.6	177	28.6	1.40	(1.0-1.9)	**0.029**
Enhanced vegetation index (EVI)							
<2500	39	5.4	139	22.5	1	-	-
2500-4499	570	78.6	453	73.3	3.69	(2.5-5.5)	**<0.0001**
≥4500	116	16.0	26	4.2	11.55	(6.5-20.4)	**<0.0001**
Temperature tertiles							
Lowest 1	313	43.2	138	22.3	1	-	-
2	200	27.6	253	40.9	0.26	(0.2-0.4)	**<0.0001**
Highest 3	212	29.2	227	36.7	0.43	(0.3-0.6)	**<0.0001**
Elevation (m)							
<960	207	28.6	266	43.0	1	-	-
960-979	284	39.2	209	33.8	0.90	(0.6-1.3)	0.570
980-1007	233	32.2	143	23.1	0.89	(0.6-1.4)	0.618
Total rainfall (mm)							
<70	105	14.5	331	53.6	1	-	-
70-114	270	37.2	186	30.1	4.32	(3.2-5.9)	**<0.0001**
115-258	350	48.3	101	16.3	10.75	(7.6-15.0)	**<0.0001**
Second season (2016)	676	87.8	447	69.7	3.12	(2.3-4.2)	**<0.0001**

[1] Estimates calculated using robust standard errors and adjusted for matching variables (age category, gender, calendar quarter), transmission season and health facility level. [2] First component, [3] At least 1 net per every 2 people in the household, [4] In the past 2 weeks, [5] Changed residence less than 6 months [6] Mobility within the past 30 days
