# Supplementary material for: Malaria risk factors in northern Namibia: The importance of occupation, age and mobility in characterizing high-risk populations
Source: PLoS One. 2021 Jun 25;16(6):e0252690. doi: 10.1371/journal.pone.0252690 (PMC8232432; doi:10.1371/journal.pone.0252690)
Supplement: S3 Table — (PDF) [file pone.0252690.s003.pdf]

S3 Table. Latent class analyses among malaria cases: probability of latent class membership and item-response probabilities within each of the six clusters

**Characteristic**		**Class 1 (5)** Young children	**Class 2 (6)** Namibian pensioners	**Class 3 (5)** School-age children	**Class 4 (3)** Students	**Class 5 (1)** Outdoor exposures	**Class 6 (3)** Agricultural exposures
Probability of latent class membership within classes		7.40%	7.01%	21.82%	13.8%	35.6%	14.4%
Agricultural worker		0.000	0.111	0.000	0.000	0.000	**0.900**
Cattle herder		0.000	0.000	0.000	0.000	**0.058**	0.000
Child		**0.982**	0.000	1.000	0.000	0.183	0.000
Student		0.000	0.000	0.000	**1.000**	**0.303**	0.000
Unemployed/Pensioner		0.000	**0.852**	0.000	0.000	**0.256**	0.000
Security guard		0.000	0.000	0.000	0.000	**0.034**	0.015
Age category (years)							
	0-4	**1.000**	0.000	0.000	0.000	0.000	0.000
	5-14	0.000	0.074	**1.000**	**1.000**	0.000	0.000
	15-59	0.000	0.000	0.000	0.000	**1.000**	**1.000**
	60+	0.000	**0.926**	0.000	0.000	0.000	0.000
Male		0.509	**0.296**	0.524	0.557	0.577	0.602
Traditional home		0.509	0.407	0.429	0.528	0.500	0.504
Tent		0.000	0.019	0.006	0.009	**0.039**	0.020
No structure		0.053	0.000	**0.095**	0.047	0.040	0.027
Slept under net		0.456	0.477	0.345	0.311	**0.273**	**0.471**
Cross-border migrant		**0.105**	0.000	0.048	0.009	0.053	**0.094**
Cross-border travel		**0.053**	0.019	0.024	0.009	0.014	**0.048**
Outdoor activities at night							
	2 or more	**0.860**	0.519	0.363	0.377	0.282	0.282
	Work (farming)	0.000	**0.204**	0.000	0.000	0.000	**0.369**
	Sleeping	0.211	0.222	0.181	0.181	0.122	**0.321**
	Playing	0.123	0.000	**0.518**	0.292	0.099	0.000
	Bite prevention	0.018	**0.000**	0.071	0.057	0.029	0.009
